# Different megafauna vary in their seed dispersal effectiveness of the megafaunal fruit *Platymitra macrocarpa* (Annonaceae)

**DOI:** 10.1371/journal.pone.0198960

**Published:** 2018-07-18

**Authors:** Kim R. McConkey, Anuttara Nathalang, Warren Y. Brockelman, Chanpen Saralamba, Jantima Santon, Umaporn Matmoon, Rathasart Somnuk, Kanchit Srinoppawan

**Affiliations:** 1 School of Natural Sciences and Engineering, National Institute of Advanced Studies, Indian Institute of Science Campus, Bangalore, India; 2 School of Geography, University of Nottingham Malaysia Campus, Semenyih, Selangor, Malaysia; 3 Ecology Lab, BIOTEC, National Science and Technology Development Agency, Pathum Thani, Thailand; 4 Institute of Molecular Biosciences, Mahidol University–Salaya, Phutthamonthon, Nakhon Pathom, Thailand; 5 Conservation Biology Program, Mahidol University Kanchanaburi Campus, Sai Yok, Kanchanaburi, Thailand; 6 Department of National Parks, Wildlife and Plant Conservation, Chatuchak, Bangkok, Thailand; Indiana University Bloomington, UNITED STATES

## Abstract

The world’s largest terrestrial animals (megafauna) can play profound roles in seed dispersal. Yet, the term ‘megafauna’ is often used to encompass a diverse range of body sizes and physiologies of, primarily, herbivorous animals. To determine the extent to which these animals varied in their seed dispersal effectiveness (SDE), we compared the contribution of different megafauna for the large-fruited *Platymitra macrocarpa* (Annonaceae), in a tropical evergreen forest in Thailand. We quantified ‘seed dispersal effectiveness’ by measuring the quantity and quality contributions of all consumers of *P*. *macrocarpa* fruit. Seed dispersal quantity was the proportion of the crop consumed by each species. Quality was defined as the proportion of seeds handled by each animal taxon that survived to produce a 2-month seedling. Megafauna (elephants, sambar deer, bears) dispersed 78% of seeds that produced seedlings, with 21% dispersed by gibbons (a medium-sized frugivore). The main megafaunal consumers displayed different dispersal strategies. Elephants were the most effective dispersers (37% of seedlings) and they achieved this by being high-quality and low-quantity dispersers. Bears displayed a similar strategy but were especially rare visitors to the trees (24% of the total seedlings produced). Sambar were high-quantity dispersers, but most seeds they handled did not survive and they were responsible for only 17% of seedlings. Gibbons displayed a high SDE relative to their body size, but they probably cannot match the role of elephants despite being more regular consumers of the fruit. The low density and poor regeneration of *P*. *macrocarpa* in the study site suggest that current dispersal rates by megafauna are insufficient, possibly reflecting reduced or missing megafauna populations. We show that different megafaunal species disperse seeds in different ways and may make unique contributions to the reproductive success of the plant species.

## Introduction

The world’s largest animals are extremely important, yet often very vulnerable, components of ecosystems [1, 2]. These animals play vital functional roles across a diverse range of habitats, with smaller animals usually lacking the capacity to replicate these functions [1, 3, 4]. However, not all functions performed by megafauna have been adequately assessed at the community level across the range of habitats that they occupy [1]. Megafauna animals are argued to be important seed dispersers because of their capacity to consume large amounts of fruit and disperse the seeds over long distances [5–7]. They are particularly important for the dispersal of very large fruit (often termed “megafaunal” fruit, which are considered to be greater than 4 cm wide [8]). This is supported by studies in drier, herbivore-dominated habitats [9–12], but there have been few studies in rain forests [13, 14] where smaller frugivores often occur in higher abundance and consequently can consume more fruit than the herbivorous megafauna, whose diets are often dominated by non-fruit items [15, 16]. Hence, smaller frugivores may rival the proposed role of megafaunal herbivores in these habitats and it is important that we compare, empirically, the seed dispersal capacity of these large, herbivorous mammals.

A second problem with determining the ecological roles of megafauna is the diverse range of animals this term often encompasses. “Megafauna” has been used to refer to the very large “megaherbivores” (>1000 kg in body mass) as well as “large herbivores” which can be an order of magnitude smaller (45–1000 kg) [1]. These animals have different behaviour and digestive systems and could be expected to differ substantially in their ecological roles in processes such as seed dispersal, which may result from the consumption of foods that are relatively minor items in their diet. Hence, “megafauna” encompass a broad collection of animals of varying functional importance in the ecosystem. Very few studies have compared dispersal capacities among the different taxa than have been labelled as megafauna [7], probably because researchers have focused on more numerous and obvious seed dispersers such as birds and primates.

Our aims in this study were to (i) determine if different megafaunal seed dispersers displayed similar dispersal effectiveness, and (ii) determine whether a tropical rain forest fruit large enough to be called a “megafaunal fruit” is dependent on megafauna for dispersal. Alternatively, smaller, more abundant frugivores might match the dispersal effectiveness of herbivorous megafauna. We studied *Platymitra macrocarpa* Boetl. (Annonaceae), in a forest supporting an intact (within current times) mammalian frugivore assemblage, with primates (medium-sized frugivores), ungulates and bears (“large herbivores”) and elephants (“megaherbivores”). We determined seed dispersal effectiveness of an animal by measuring both its quantitative and qualitative components [17]. Quantity was measured as the proportion of the crop consumed by each taxon. In this study, quality was measured as the likelihood of a consumed and egested or defecated seed surviving and producing a two-month old seedling.

## Materials and methods

We combined multiple datasets collected in, or adjacent to, the Mo Singto Forest Dynamics Plot, Khao Yai National Park, Thailand, to study the different stages of the seed dispersal process: frugivory in the canopy, frugivory by terrestrial animals, and seed fate (Table 1). The main study of seed dispersal (in the canopy and on the ground) was carried out in four trees from May 2015 to August 2016, with seed fate monitored until December 2016. Opportunistic data on dispersal by elephants and macaques, collected in previous years both on and off the Mo Singto plot (2011 and 2014), were also used. Phenology data (2009–2016) from the Mo Singto Plot is also used [18].

**Table 1:** Summary of methods used to assess seed dispersal effectiveness.

Seed dispersal stage	Methods used	Details	Sampling effort
Frugivory in canopy	Transects under tree	To quantify proportion of fruit handled by different consumers and proportion uneaten and available to terrestrial consumers	3 trees each monitored over 14 days; 1 tree monitored for 4 days
	Direct observations	To identify consumers and their handling signs	16 h across 4 trees
Frugivory on ground	Camera-traps	To identify terrestrial consumers and estimate proportion of fruit handled by each species	102 camera-trap days
	Fruit and seed fate monitoring	To determine proportion of fruit taken by terrestrial consumers and proportion left uneaten	85 whole fruit monitored for 7 months (across 3 trees)
Dispersal distances (under vs. away)	Transects under tree	To determine the proportion of seeds handled by arboreal consumers that were not found under the tree (and were therefore dispersed away from the crown)	3 trees each monitored over 14 days; 1 tree monitored for 4 days
	Opportunistic data	Sambar deer were determined to regurgitate seeds based on seeds found at a bedding site and direct observation of a habituated sambar feeding on *Platymitra* fruit	7 fruit fed to deer; 1 bedding site found with 5 regurgitated seeds
	Previous knowledge	Deer and elephants were assumed to disperse all seeds away from the crown based on established knowledge of their behaviour	
Seed fate under crown	Fruit and seed fate monitoring	To determine germination rate of seeds dispersed under tree crown	85 whole fruit; 66 partly-eaten fruit; 58 seeds monitored across 3 trees (for 7 months)
Seed fate away from crown (no dung)	Seed fate monitoring (experiment)	To determine germination rate and survival of seedlings for seeds dispersed away from tree crown	100 seeds monitored along 2 transects (for 7 months)
Seed fate in dung	Monitoring of seeds in elephant dung	To determine emergence and survival of seedlings in dung	91 dungs searched, but only 1 found with *Platymitra* seeds (n = 75 seeds) that could be monitored.

Permission to conduct the research was granted by the National Research Council of Thailand with the consent of the Department of National Parks, Wildlife and Plant Conservation. Research on vertebrate animals was non-invasive and involved direct observations, camera trapping (both explained below) or a secondary description of feeding signs. Hence, approval from an animal ethics committee was not required according to BIOTEC and Department of National Parks regulations.

### Study site

The study site was the 30-ha Mo Singto Forest Dynamics Plot (101°22′ E, 14°26′ N [19]) in Khao Yai National Park, central Thailand. The plot, located at 725–815 m altitude, lies in seasonal evergreen forest that receives 1200–3000 mm of rainfall per year (average about 2100 mm over 21 years), mostly during May–September, with a dry season from October to April. All woody trees and shrubs with dbh ≥ 1 cm have been mapped, tagged, and identified on the plot [20]. The phenology of 60 common species on the plot (including *Platymitra macrocarpa*) has been scored twice per month since 2003. Maximum fruit availability occurs from April to June and sometimes in August [20]. The park (2168 km^2^) supports a diverse bird (320 species) and mammal fauna (at least 71 species) [21].

### Study animals

Using the definitions for megafauna given by Malhi et al. [1], the Mo Singto plot has one megaherbivore (Asian elephant, *Elephas maximus*: 3500 kg) and three large herbivores (Sambar deer, *Rusa unicolor*: 180 kg; Asiatic black bear, *Ursus thibetanus*: 109 kg; sun bear, *Helarctos malayanus*: 53 kg) that consume fruit and can disperse seeds. Elephants are reasonably common within KYNP [22]. Over 13 months (August 2015–2016) the elephants entered the plot seven times, mostly in August and January. No abundance data exist for other resident megafauna, but sambar were commonly observed in adjacent grasslands during the study and regurgitated seed piles in the forest, indicating their use of this habitat. Gaur (*Bos gaurus*) is also present in KYNP, but visit the plot only rarely.

Four medium-sized frugivores present on the plot also consume *Platymitra macrocarpa* fruits (muntjac, *Muntiacus muntjak*: 25 kg; macaques, *Macaca leonina*: 10 kg; gibbons, *Hylobates lar*: 6 kg; black giant squirrel, *Ratufa bicolor*: 1.5 kg). Gibbons are generalist frugivores that occur at high density, with about four groups (16 individuals) per square kilometer in the Mo Singto study area [23]. They are highly effective seed dispersers [19]. Pig-tailed macaques occurring on the plot occupy a much larger range (449 ha [24]) and enter the plot frequently in a group of over 50 individuals. There is no information available on the abundance of giant squirrels and muntjacs. No birds that can consume such large fruits (e.g., parrots [25]) occur at the study site.

### Phenology and fruit characteristics

Flowering and fruiting in seven *Platymitra macrocarpa* trees on the Mo Singto plot have been recorded twice monthly since 2009, providing seven years of data [18]. In 2015, 11 of 13 trees on the plot with dbh >15 cm were also checked for fruiting status. We recorded fruit length, width and breadth, and seed number, for 26 fruits from multiple trees. We also recorded seed length, width and breadth for seeds (n = 30) taken directly from fruit, from elephant dung or from seeds regurgitated by sambar; since these did not differ significantly in size from seeds not swallowed by sambar we present combined data.

### Seed dispersal and seed fate

We studied four fruiting trees which included two trees on the plot (the only trees on the plot found to have fruit) as well as two fruiting trees near the edge of the plot that were in similar mature or old-growth forest. Four types of data were collected from these trees in 2015: direct observations, seed dispersal, fruit and seed fate along transects (Table 1). Nocturnal observations of terrestrial visitors from camera traps and seedling transects (in 2016) were collected from three trees (since the fourth tree had a broken crown).

#### Direct observations of arboreal animals

Given the low visitation rates of arboreal frugivores (primates and squirrels), we did not conduct systematic tree watches and obtained fruit consumption data by examining fruits or fruit parts falling on transects. However, we completed 16 h of opportunistic watches at the four trees in May 2015, to verify the feeding sign we recorded along transects. These data were not used in the estimation of fruit consumption. Watches were done from 0815 h to 1400 h while checking transects under the trees. Since primates and squirrels (only *Ratufa bicolor* was observed) are partly habituated to observers on the Mo Singto Plot [26], it is unlikely that our presence deterred feeding.

#### Transects under tree: Frugivory and seed dispersal by arboreal consumers

To determine the proportion of fruit handled by different arboreal consumers (primates and squirrels) and the proportion of uneaten fruit falling to the forest floor (and therefore available to terrestrial consumers), we monitored fallen fruit and seeds and feeding signs along four transects located under the canopies of the four trees. Transects radiated N, S, E, W from the tree base to the edge of the canopy and were 1 m wide. The radii of the canopies (hence transect length) ranged from 6 to 12 m, and averaged 9 m.

Transects were monitored every 1 to 2 days for 2 weeks, and all fallen whole fruit, partly-eaten fruit and seeds were recorded. Distinctive feeding signs on fruits and seeds allowed us to distinguish the frugivores: primates (macaques and gibbons) leave tooth impressions in the pulp and husk that are different from the chisel-like marks left by squirrels [27]. Fruit with claw marks were determined to have been consumed by bear (species not determined). We could not distinguish fruits that had been partially eaten by macaques from those consumed by gibbons; however, because macaques spit the large seeds and gibbons swallow them, we assigned feeding events to macaques when spat seeds were found along transects. To determine total number and proportions of fruit being handled by the consumers we multiplied the density of fallen fruit and seeds by the total crown area (mean across four trees was 254 m^2^).

Determining the proportion of seeds dispersed away from the tree crowns by the arboreal consumers is important for assessing their effectiveness. We trialed the use of transects away from the crown to measure dispersal distances, but these were unsuccessful due to the steep terrain and low visitation rate. Hence, we simply determined the proportion of seeds dispersed under vs. dispersed away from the tree crown. To do this we counted the number of handled fruits by each arboreal consumer (from the inedible portions remaining), and the number of seeds each consumer dropped or spat along the transects under the tree crowns. Using an average number of 7 seeds per fruit, we estimated the number of seeds that were not found along the transects and that were assumed to have been swallowed or carried away. We believe it unlikely that entire fruits were carried away from the tree crowns (and therefore not recorded along the transects) by arboreal consumers, as they consistently only partially consumed fruit, dropping half the seeds within the fruit under the tree crown. Only the semi-terrestrial macaques might have been able to carry the heavy fruits, but this is probably a rare event given the infrequency of their feeding visits and their known behavior [26, 34].

#### Camera traps: Frugivory by terrestrial animals

Frugivory by terrestrial animals (elephant, sambar and muntjac) was measured using camera traps. Eight cameras were placed under three of the study trees for between 4 and 39 days for a total of 102 camera-trap days. The cameras were set to take three photos at each trigger release. The cameras recorded 1119 photos of 51 independent animal visits; 8 of these visits were by animals that showed no signs of fruit consumption (rat, variable squirrel, common palm civet) and these were excluded from the results. Sambar, elephants, macaques and muntjac (*Muntiacus muntjak*) were all recorded foraging and all their visits were recorded. We noted the number of minutes that were recorded by the cameras for each feeding event (estimated from the times of the photo captures) and were also able to estimate the time it took for these species to consume an individual fruit.

To compare frugivory by terrestrial animals with that of arboreal animals it was necessary to estimate the proportion of fruits consumed by terrestrial animals. The fruit falling to the ground uneaten (calculated from frugivory transects above) estimated the total quantity available to terrestrial animals. We measured the disappearance rate of these fruits (fruit and seed fate methods explained below) and used this value as the total proportion of fruit handled by terrestrial animals. We then calculated the number of minutes each species was recorded on the cameras multiplied by the feeding rate (fruits eaten per minute); the relative proportions of fruit taken by elephants, sambar and muntjac were then used to determine what proportion of fruit that was taken from the ground could be assigned to each animal. We could not obtain a feeding rate for muntjac so we arbitrarily assigned them a rate that was half that of sambar (5x the weight of a muntjac). Based on established knowledge it was assumed that all these animals dispersed seeds away from the crown of parent trees [12].

#### Opportunistic data on sambar deer

Deer can be seed predators or seed dispersers but obtaining data on them is difficult. Hence, we obtained opportunistic data on sambar to determine seed handling. In 2015 we offered *P*. *macrocarpa* fruit to a male sambar whose bedding site was close to human habitation. Because this individual ranged freely in the forest, we could not systematically record regurgitation times and recovery rates. The deer ate all seven fruits offered (approximately 49 seeds). It consumed the entire fruit but spat 12 seeds (24%). Five seeds (17%) were regurgitated at the bedding site between 6 and 11 h after consumption; the remaining ca.32 seeds were not found. The regurgitated seeds had a distinctive smell which helped us to identify seeds regurgitated by sambar in the forest. In the forest, we found a single bedding site of a sambar (identified by the size of hoof imprints and smell) that had 5 regurgitated *P*. *macrocarpa* seeds. Sambar bedding sites can be suitable places for seed germination [5] and, therefore, we confirmed that sambar were seed dispersers of this species.

### Fate of seeds

#### Seed fate under tree crowns

To determine the fate of seeds under the parent tree crown, all fallen fruit, partly-eaten fruit and seeds found along or close to the frugivory transects (described above) were marked with wooden chopsticks and flagging tape. We aimed to obtain at least 20 of each type (whole fruit, partly-eaten fruit, seed) under each of three study trees (the crown of the fourth tree was broken and was not used to estimate seed fate). The fates of dropped seeds and fruits were recorded every 1–2 days for the first month (May to June), and then monthly for six months (until December).

#### Seed fate away from tree crowns for seeds not in dung

Macaques and deer potentially disperse seeds away from tree crowns via spitting and regurgitation. To determine the fate of these seeds, we monitored 100 seeds taken from whole fruit and cleaned of all pulp. These were placed along each of two trails that were at least 50 m from a fruiting *P*. *macrocarpa* (n = 20 treatment locations, 10 on each transect). At each 25-m point along the trails, two groups of 5 seeds were placed on either side of the trail–separated by at least 5 m; 5 seeds represents the number found at the sambar bedding sites. The fate of these was recorded every 2–4 d for 3 months, and then monthly for 4 months. Seeds that remained each month were inspected for insect holes.

#### Seed fate away from tree crowns for seeds in dung

Between 2014 and 2016 we searched 91 elephant dung piles for *P*. *macrocarpa* seeds on or close to the Mo Singto plot. When seeds were found we continued to monitor them every two weeks to determine time of seedling emergence. We could not obtain seed fate information for seeds dispersed in the scats of bear and gibbons due to the rarity of finding their scats; since dung can have a strong impact on seed fate we used seedling survival rate of seeds in elephant dung as a proxy for those dispersed by bears and gibbons when calculating final seed dispersal effectiveness of the different animals.

### Seedling establishment

We conducted searches for 1-y old seedlings (germination is at approximately 4 mo) along the same transects used to monitor frugivory and seed fate. Seedlings were counted to confirm if they could establish under and away from the crown, but these were not included in measurements of SDE since the seedlings could not be associated with a disperser. The seedling transects were done 15 mo after the start of the study (in August 2016) and for only 3 trees (the fourth tree had a broken crown). Two of these trees were fruiting again. The transects were 1 m wide and extended from the tree base to the end of the crown, in 4 directions (N, S, E, W). Total crown area searched across the 3 trees was 103 m^2^ (34–35 m^2^ each tree). We established additional transects to search for seedlings away from the crown as well and these were sampled once. These were 2 m wide (to provide a greater search area) and radiated from the “under” transects for an additional 30 to 50 m beyond the tree crown (distance depended on terrain and obstructions such as tree falls). The total distance searched away from the crowns was 750 m^2^ (220–300 m^2^ per tree).

### Seed dispersal effectiveness

Seed dispersal effectiveness (SDE) was determined as the product of a quantity and quality component (SDE = quantity x quality; Schupp et al. [17]), and was measured for each seed-dispersing animal and for uneaten fruits. The quantity component was the proportion of the crop handled by each animal species as described in the previous sections. In this study the quality component was defined as the proportion of seeds handled by each animal species that produced a seedling that persisted for at least 2 months (based on the results from our seed fate data). To calculate the quality component we applied seedling emergence/survival rates from the seed fate experiments to the proportion of seeds dispersed under vs. away by the different animal species, and used the combined value of both of these; this was only applicable to gibbons and macaques which deposit seeds both under and away from the tree crown. Gibbons disperse seeds in scats that scatter after falling to the ground, hence not all are contained in dung after deposition. Around 10% of seeds this size are buried by dung beetles [27] and we calculated the seedling establishment rate by applying the rate for seeds in dung to buried seeds, and applying the rate for seeds not in dung to the unburied seeds.

## Results

### Phenology and fruit traits of *Platymitra macrocarpa*

**Fig 1:**
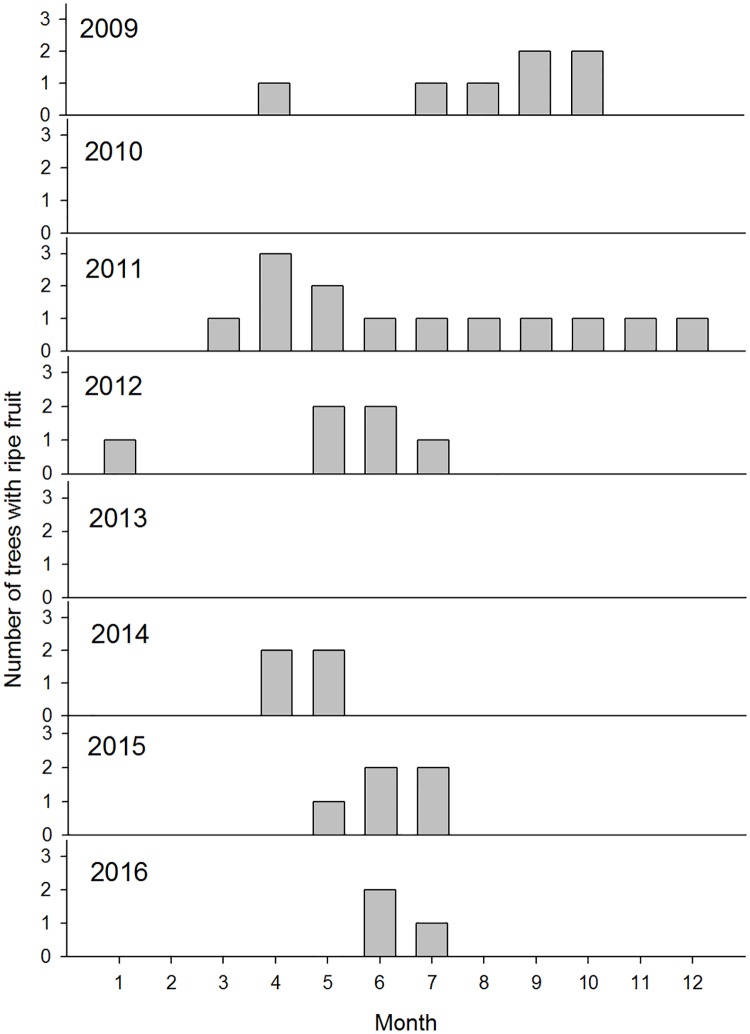
Number of *Platymitra macrocarpa* trees producing ripe fruit over seven years of phenological monitoring. A maximum of 3 trees (out of seven monitored trees) fruited in any year.

Of the seven trees monitored for phenology, only two regularly had fruit (in five and six of the eight studied years, respectively) and four trees never produced fruit (Fig 1). Only trees above 35 cm dbh produced fruit and the regular fruiters (producing fruit in 20–45% of months) were larger than 55 cm dbh. Ripe fruit tended to be available from May to August, but this varied among years (Fig 1). An individual tree had ripe fruit for 6.1 mo (mean; range 3–20 mo; n = 2 trees with dbh > 50 cm) during each fruiting episode.

The brown fruits of *P*. *macrocarpa* measured 80 x 60 x 58 mm on average (n = 22) and had a mean of 7.6 seeds (range: 5–10) (S1 Table). Fruits measured in 2014 from one of the same trees averaged 123 x 89 x 85 mm (n = 2), suggesting considerable size variation across years. Seeds measured 30 x 18 x 11 mm (n = 30) on average and were each surrounded by a thin, firmly-attached layer of juicy-soft pulp that is typically attractive to macaques and gibbons (and was swallowed by them). Surrounding the seeds and pulp was dry, medium-soft tissue that hardened after approximately 3 days on the forest floor; this tissue was consumed by sambar and, presumably, elephants but was discarded by primates.

### Frugivory and seed dispersal by arboreal animals

**Fig 2:**
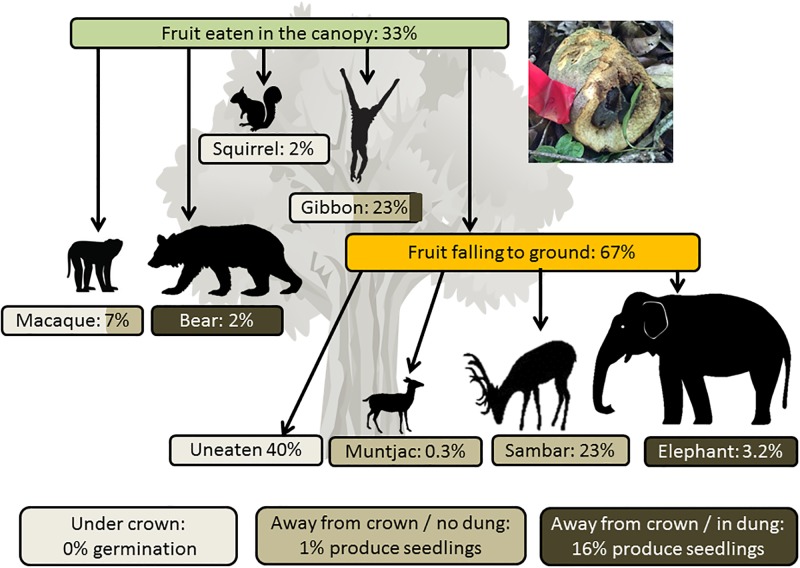
Quantity of fruit eaten by different consumers of *Platymitra macrocarpa*. The image of a partially consumed fruit shows the soft dry outer part eaten by terrestrial consumers, and the seeds covered by juicy soft pulp that is consumed by the arboreal consumers.

**Fig 3:**
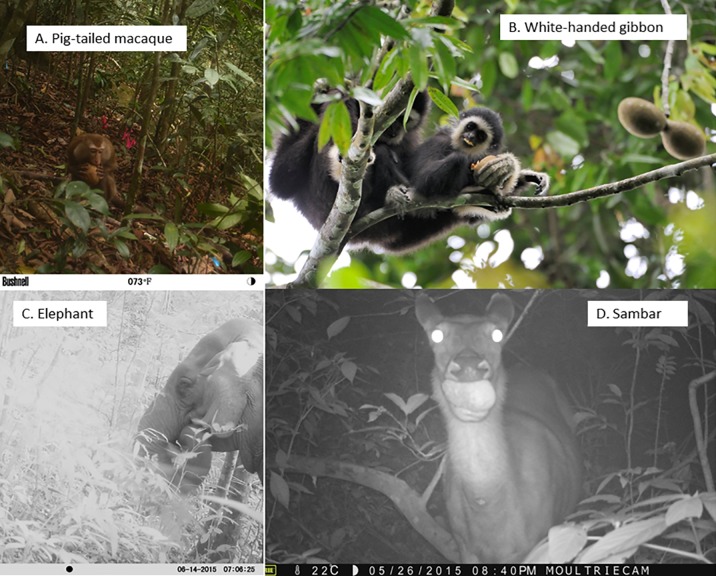
Major consumers of *Platymitra macrocarpa*. A. Pig-tailed macaque biting into a whole fruit; B. White-handed gibbon consuming fruit in the canopy (Photo by Kulpat Saralamba); C. Elephant placing a fruit in its mouth; D. Sambar consuming a whole fruit.

In the 16 h of tree watches, we observed gibbons feeding at *P*. *macrocarpa* trees for 10 min. Two giant black squirrels and one macaque were observed close to the trees, but did not feed. The estimated daily crop size of the *P*. *macrocarpa* trees was 64 ± 11 ripe fruits/day (mean ± SE across 4 trees (Table 1)), and most of these fruits fell to the ground uneaten (67 ± 9%) (Fig 2, S2 Table). Arboreal animals (gibbons, macaques, squirrels) consumed 33% of fruit. Gibbons were the most commonly recorded frugivore (Fig 3), consuming 23 ± 7% of available fruits; they consistently partially-consumed the fruits, dropping half the handled seeds to the forest floor (still attached to the outer rind), with the remainder swallowed and dispersed away from the parent crowns (no feces was found along the transects). Macaques consumed 7 ± 4% of fruits available in the canopy (and also consumed fruit terrestrially; see next section). They also partially consumed fruit (dropping half of the handled seeds), and also spat seeds under the parent crowns; only 25% of handled seeds were estimated to be dispersed away from the tree crown region. Squirrels consumed 2 ± 2% of fruits and dropped all seeds beneath the crown. A bear handled 2 ± 2% of fruits and all seeds were removed from the fruit (and presumably swallowed). We cannot confirm if the fruits consumed by bear were obtained on the ground or from within the canopy, but the handled fruits were found along the transects used to document feeding by arboreal animals.

### Frugivory by terrestrial animals

Sixty-seven percent of the fruit crop (SE = 9%, n = 4 trees) was not consumed by arboreal frugivores and fell to the ground whole (Fig 2, S3 Table). Terrestrial animals consumed 40% of the fruit available on the ground or 27% of the total crop. Fresh fruit was removed by terrestrial animals 1–17 days after falling to the forest floor (7.4 ± 1.0, mean ± 1 SE, n = 29). The cameras recorded 1119 photos of 51 independent animal visits, and 43 of these visits were by foraging animals. Sambar deer were the most commonly observed animals with 29 independent observations (81% of the minutes in which foraging animals were observed, and consumed fruit at a rate of 2.3 per min, n = 4 observations) (Fig 3). Macaques were observed on seven occasions (9% of minutes) and elephants four times (8% of minutes, consuming 4 fruits per minute in 2 observations). Barking deer were observed the least (3 visits, 2% of minutes; they were arbitrarily assigned a feeding rate of ½ that of sambar to allow SDE to be calculated). Using these values we calculated the percentage of the total crop that each terrestrial animal handled (Fig 2).

### Fate of seeds

#### Seeds under the tree

**Fig 4:**
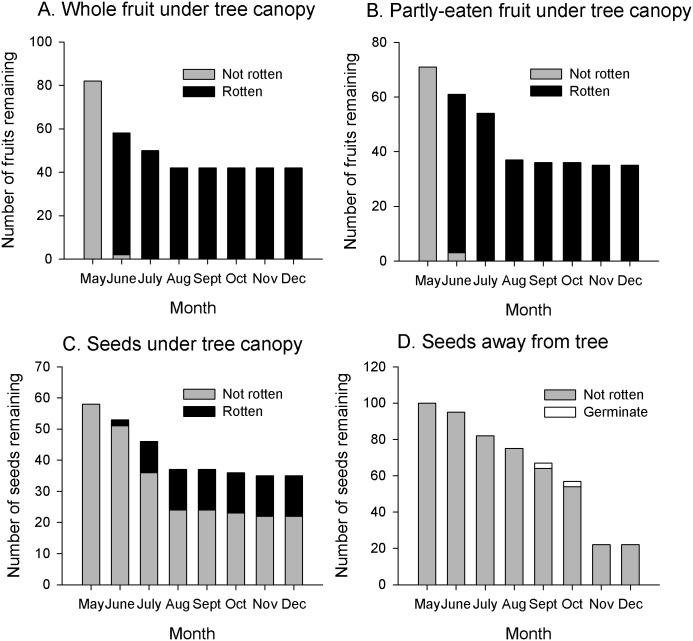
Fate of *Platymitra macrocarpa* fruit and seeds present on the forest floor. (A) whole fruit, (B) partly-eaten fruit, (C) seeds under source canopy and (D) seeds away from the source. Shown are the numbers of fruits or seeds that remained in-situ each month and, of those, how many were noted to be rotten or that germinated over the 6-mo sampling period. Sample sizes for each treatment differ and are shown by the May bar on the graphs.

No seeds from whole fruits, partly-eaten fallen fruits or seeds monitored under fruiting trees germinated (Fig 4, S4 Table). All fruits and seeds that remained beyond one week were infested by bruchid beetles (genus unknown) within 8–11 d. All whole and partly-eaten fruit, and seeds under the fruiting trees, disappeared (recorded as “gone”) prior to August (Fig 4A, 4B and 4C), which is approximately when fruit became less available on the tree. Many of the seeds under the tree were also rotten at this time. Ultimately, these results suggest that fruits and seeds deposited under the crown that did not disappear have little or no chance of establishing seedlings; however, 49% of whole fruit and 51% of partly-eaten fruit were recorded as “gone”. We have confirmation from the cameras that whole fruits were removed by terrestrial frugivores, but have no photographic evidence that partly-eaten fruits were consumed by these animals. Four independent visits by a rodent were recorded by the camera traps but the animals were not interacting with seeds or fruit; hence we cannot confirm if seed hoarding rodents removed seeds.

#### Seeds away from the tree

Of the 100 seeds monitored at least 50 m away from a fruiting *P*. *macrocarpa* tree, 3% germinated at 4 mo but all the seedlings had died by 3 mo (S5 Table). Seeds were infested by beetles within 6–22 d, and no seeds away from the crown became rotten. Removal of seeds on the ground away from the crown was more steady over the months than seeds under the crown, with a peak in November (Fig 4D).

#### Seeds in dung

Of the 91 elephant dungs searched, 3 contained *P*. *macrocarpa* seeds in the quantities 1, 71, 75. Only one of these dungs (n = 75 seeds) could be monitored for seedling emergence as the others were on a road. Twelve seedlings (16%) emerged 10 months later (August 2016).

### Seedling establishment

Very few seedlings were found under or away from the tree canopies (n = 3 trees, S6 Table), one year later and the results noted here are for all trees combined. In total, 5 seedlings were found in the 103 m^2^ checked under three trees, giving a density of 0.05 seedlings m^−2^. Three seedlings were found in 750 m^2^ of transects away from the crowns giving a density of 0.004 seedlings m^−2^; one of the seedlings was at 24 m, and two within 5 m of the crown edge.

### Seed dispersal effectiveness

**Fig 5:**
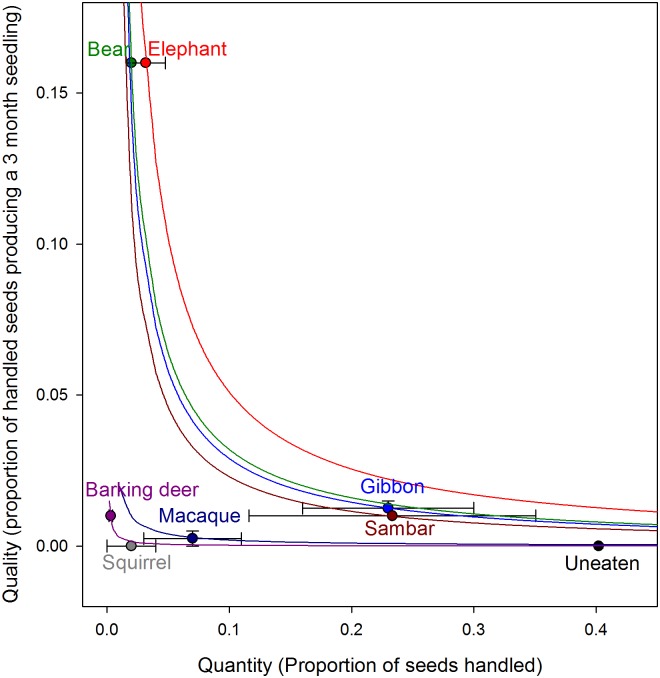
Seed dispersal effectiveness (SDE) landscape, showing the seven consumers of *Platymitra macrocarpa*. The circles indicate the quantitative and qualitative components, and the isoclines show the seed dispersal effectiveness (quantity × quality) of the consumers. The error bars represent the SE values.

Elephants were the most effective of *P*. *macrocarpa’s* seed dispersers (SDE = 0.0051; 37% of seedlings) (Fig 5); while their SDE was only marginally higher than that of the next group of animals, even at their maximum recorded value (as noted by the SE bars on the figure) these animals could not match the effectiveness of elephants. Bears (SDE = 0.0032; 24% of seedlings), gibbons (SDE = 0.0029; 21% of seedlings) and sambar deer (SDE = 0.0023; 17% of seedlings) were the next most effective dispersers. These animals achieved similar SDE values in different ways, however. Sambar deer and gibbons were high quantity but low quality dispersers for this species, while bears consumed few fruits but through the deposition of seeds in dung were assumed to have a higher quality role. Macaques (SDE = 0.0002; 1% of seedlings) and barking deer (SDE = 0.00003; 0.2% of seedlings) were ineffective dispersers, while seeds dispersed by squirrels or contained in whole fruit never produced seedlings.

**Fig 6:**
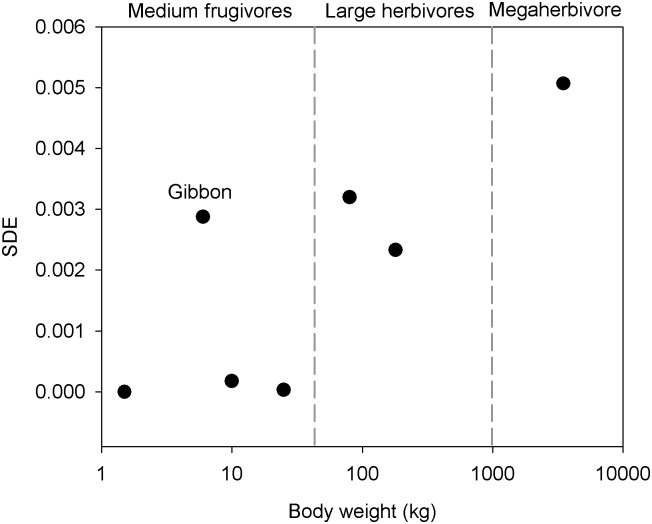
The relationship between seed dispersal effectiveness (SDE) and body weight of the seven consumers of *Platymitra macrocarpa*. The consumers are divided according to their diet group.

There was a general decrease in SDE values as body weight of the disperser species decreased (Fig 6), although this relationship was not significant (Spearman Rank, *r* = 0.679, *P* = 0.09). Most of the medium-sized frugivores were not effective dispersers, with gibbons as an anomaly having similar effectiveness as the large herbivores.

## Discussion

Seeds of the megafaunal fruit *Platymitra macrocarpa* were dispersed mostly by megafauna (78% of seedlings) in a tropical evergreen forest in Thailand. However, the megafaunal dispersers had very different dispersal strategies, with the large-herbivore dispersers (sambar deer and bears) unable to replicate the role of the megaherbivore (elephant). Indeed, the decline in SDE values with body size of most dispersers confirms that the different animals often referred to as megafauna can differ substantially in their seed dispersal roles. The most effective disperser of this megafaunal fruit was the only “true” megafauna, the elephant. Elephants were responsible for 2× the seedlings of sambar and 1.5× the seedlings of bears; although they were uncommon visitors to the trees they could consume large quantities of fruit when present [6] and they also deposited the seeds in large dungs where they had the highest likelihood of survival. Sambar deer, on the other hand, were nightly visitors to the trees and consumed more than three times as much fruit as most other animals, including elephants. However, the high rate of seed predation by beetles meant that ultimately, seeds dispersed on the forest floor by sambar had a very low chance of survival. Bears (sun bear or Asiatic black bear) were rare visitors to the trees but because they also deposit seeds in large fecal masses [30, 31] they are potentially effective as seed dispersers for this species. However, we could not monitor seed survival in bear dung and have conservatively assigned them a survival rate identical to elephants, which could inflate their importance.

Despite the large size of this megafaunal fruit, and the relatively small size of gibbons, these frugivores had SDE values that rivalled that of the large herbivores. Gibbons are one of the most common frugivores and effective seed dispersers in this forest [18, 26] and were one of most prevalent consumers of *P*. *macrocarpa*. Although they dispersed the seeds effectively they may be unable to maintain the recruitment of this species. Most seeds defaecated by gibbons scatter after landing on the forest floor, and only around 10% of seeds this size are buried by dung beetles where they have a chance to avoid beetle infestation [29]. Hence, the majority of seeds will not produce seedlings, making *P*. *macrocarpa* apparently reliant on megaherbivores for its dispersal. Indeed, the recruitment of this species is poor on the Mo Singto Plot. Rates of seedling establishment along the monitored transects were low (40 seedlings ha^−1^), and *P*. *macrocarpa* has a relatively low ratio (4.6:1) of young trees of pre-reproductive size in relation to larger trees (median value across all trees is ca. 7:1; [20]). The tree is also uncommon in the forest with a density of 3.9 individuals ≥1 cm in dbh ha^−1^ (“rare” species occur at densities <1 indiv ha^−1^, and “common” species ≥10 indiv ha^−1^ [20]). *P*. *macrocarpa* appears to be reliant on megaherbivores for its dispersal, and the current low regeneration rate could reflect a decline in megaherbivore numbers in the park.

In relatively recent times, the rainforests of Thailand were inhabited by two megaherbivore taxa–rhinoceros (Sumatran and Javan) and elephant. Almost no information exists regarding the seed dispersal capacity of a forest rhinoceros, and their populations exist only as relics, having disappeared from almost all of their former ranges [32]. However, forest rhinoceroses are considered primarily browsers, compared to the grazing habits of Asian elephants, and there is anecdotal evidence for them playing a role in seed dispersal [12, 33]. It is possible that the ‘gap’ in seed dispersal that appears to exist for this megafaunal fruit species is an indication of the disappearance of an unknown former disperser. This gap might also reflect a reduction in elephant movements in recent years around the Mo Singto Plot, which is near park headquarters whose development has blocked some elephant routes, although the population itself is considered healthy [18, 21]. More fruit of this species and another megafaunal fruit (*Garcinia benthamii* [27]) goes undispersed (both >40%) than other, primate-dispersed, fruit species (<5% to 25%; [26, 27, 34]) in this forest. This is reminiscent of the “anachronistic fruit” in Neotropical habitats, where the putative megafaunal dispersers became extinct thousands of years previously [8, 35] and might be the present and future reality of many megafaunal plant species in Asia.

Megafaunal fruits are described by Guimarães et al. [8] as being between 4 and 10 cm wide with up to five seeds (Type 1), or being larger than 10 cm wide with many small seeds (Type 2). *P*. *macrocarpa* fits this size definition although the fruit has more than five seeds on average. This species is also dull in colour (brown) as predicted for a megafaunal fruit (Guimarães et al. 2008) and is not a fruit preferred by arboreal mammals which are major seed dispersal agents in this habitat [26]. Two other traits of this species also make it most suitable for megafaunal dispersal; first, the long fruiting period of approximately six months (and up to 20 months for one tree), and the steady rate of falling fruit (14 fruits day^−1^), ensured that fruit was available over a long time period and had a higher chance of being available to the irregular passing of elephants. Elephants move over vast distances so may revisit areas only across large time scales, but have a high capacity for consumption when they encounter a food source [6]. Second, seeds dispersed within dung had a much higher survival rate to the second month (16% compared with 1%) and were, ultimately, the only seeds that had seedlings surviving beyond one month.

Given the irregularity with which elephants fed on *P*. *macrocarpa*, and the difficulty in locating seeds regurgitated by sambar or defecated by gibbons, our seed fate results are short on replication; however, we believe that some meaningful conclusions are possible. The seeds were heavily targeted by beetles, making seed burial nearly essential for survival [28]. The relatively high survival of seeds in elephant dung could be an effect that significantly boosts the quality of dispersal by elephants. Interestingly, the seeds in elephant dung took much longer to germinate (10 mo) than seeds on the forest floor (4 mo) and this could be responsible for the increased seedling survival. Seedlings produced at four months must persist through the drier, cool, winter period, whereas most seedlings produced at 10 months emerge during the warmer wet season [19]. It is possible that damage by beetles to the endosperm triggers faster germination time [36], ultimately leading to lower survival. Our results therefore suggest that a major advantage of dispersal by elephants is that it delays germination until the arrival of conditions that favor seedling survival.

Seed caching by rodents could also alter seed survival rates, and we were unable to determine if this occurred in our system. We recorded very few rodents foraging under the *P*. *macrocarpa* trees suggesting that rodent consumption and hoarding were not major events. On the other hand, many seeds disappeared and the timing of seed removal coincided with periods of maximum caching in other regions [37]. Scatter-hoarding has not been investigated in this study system so we can make no conclusions of the importance of this for *P*. *macrocarpa*. A third limitation with our study was the low sample size of four trees, of which only three were studied intensely. While including more trees would have improved the accuracy of recording rare events, such as visits by bears and elephants, we did monitor all fruiting trees within a region greater than 30 ha. The low sample size reflects the rarity of this megafaunal fruit species.

## Conclusions

The world’s remaining megafauna play important functional roles in the increasingly threatened habitats that dominate Earth today [1]. Often we assume that given their size and dominance they must be important, but evidence from body size is not always the strongest determinant of functional importance [38]. Our results here suggest that highly frugivorous, smaller dispersers can disperse megafaunal fruit species effectively, but may lack the capacity to maintain their regeneration, indicating an important role for megafauna for some plant species. However, the dispersal strategies exhibited by mega-herbivores (elephants) and large herbivores (deer and bear) were very different, indicating that it is essential we do not over-generalize the role of “megafauna”, and consider their unique contributions. If less vulnerable large herbivores are unable to replicate the seed dispersal role of threatened mega-herbivores, megafaunal fruit could suffer range contractions, affecting forest community composition and potentially forest carbon stocks [39,40]

## Supporting information

S1 Table Measurements of *Platymitra macrocarpa* fruits and seeds in 2015. Fruit and seeds are taken from four fruiting trees found on and nearby the Mo Singto Dynamics Plot, Khao Yai. Seeds were taken from whole fruit, or found in elephant dung or were regurgitated by sambar.
(XLSX)

S2 Table Number of fruit, partly-eaten fruit and seeds found along transects under four fruiting trees of *Platymitra macrocarpa*, in Khao Yai National Park. Transects were 1 m wide and of varying length to match the crown spread of each tree; there were 4 transects per tree. Transects were checked every 1–2 days. Data are recorded alongside the check date. No checks were done on blank dates.
(XLSX)

S3 Table Table A. Details of where cameras were placed, dates they were placed and photos captured. Table B. Animals captured by camera traps under fruiting *Platymitra macrocarpa* trees.
(XLSX)

S4 Table Fate of whole fruit, partly-eaten fruit and seeds monitored under fruiting *Platymitra macrocarpa* trees, Khao Yai National Park. Fallen and dropped fruit and seeds were monitored under 3 trees for 7 months.
(XLSX)

S5 Table Fate of *Platymitra macrocarpa* seeds away from parent trees, Khao Yai National Park. Seeds were monitored in groups of 5 along two trails, 25 m apart and at least 50 m from the closest fruiting con-specific.
(XLSX)

S6 Table **Table A. Count of seedlings under and away from three *Platymitra macrocarpa* trees**. Trees were checked in 2016, one year after fruiting. Distances are in m. **Table B. Dimensions of transects walked**. Values are Length x Width and are given in m.
(XLSX)
